# Repetitive Element DNA Methylation is Associated with Menopausal Age

**DOI:** 10.14336/AD.2017.0810

**Published:** 2018-06-01

**Authors:** Sha Lu, Zheng Niu, Yueming Chen, Qiaofeng Tu, Yue Zhang, Wenli Chen, Wenjuan Tong, Zhifen Zhang

**Affiliations:** ^1^Department of Obstetrics and Gynecology, the Affiliated Hangzhou People’s Hospital of Nanjing Medical University, Hangzhou, China; ^2^Department of Obstetrics and Gynecology, Hangzhou Women’s Hospital (Hangzhou Maternity and Child Health Care Hospital), Hangzhou, China; ^3^Laboratory of Gene Diagnosis, the Affiliated Hangzhou People’s Hospital of Nanjing Medical University, Hangzhou, China; ^4^Department of Obstetrics and Gynecology, the Second People’s Hospital of Tonglu, Hangzhou, China

**Keywords:** DNA methylation, Repetitive elements, menopausal age

## Abstract

To investigate associations between the age of menopause and the DNA methylation levels of two repetitive elements, Alu and LINE-1, we performed plasma DNA extraction on 161 subjects and serum cell-free DNA extraction on 120 subjects. We grouped women by menopausal age as follows: ≤ 48 years (earlier menopause), ≥ 52 years (later menopause), and 48-52 years (control). The DNA methylation levels of Alu and LINE-1 were measured by MethyLight PCR. The results showed that the DNA methylation levels of both Alu and LINE-1 were inversely correlated with menopausal age in the plasma DNA cohort (r = 0.079, *P* < 0.001 for Alu; r = 0.045, *P* = 0.007 for LINE-1) as well as in the serum DNA cohort (r = 0.087, *P* = 0.001 for Alu; r = 0.041, *P* = 0.026 for LINE-1). Alu methylation levels in both the plasma and serum DNA cohorts and LINE-1 methylation levels in the plasma cohort were remarkably higher in the earlier menopause group than in the later menopause and control groups (*P* < 0.01 and *P* < 0.05, respectively). In the serum DNA cohort, the LINE-1 methylation levels in the later menopause group were significantly lower than that in the earlier menopause group and control group (*P* < 0.05). Therefore, methylation levels of Alu and LINE-1 were significantly associated with menopausal age. Women with earlier menopause showed hypermethylation in both repetitive elements, while women with later menopause showed hypomethylation. These findings suggest that altered DNA methylation in leukocytes and serum cell-free DNA may represent a biomarker of menopausal age.

Female mammals experience a gradual midlife decline in reproductive performance with age, concluding in menopause [1]. The timing of menopause onset in human beings has been linked to susceptibility to age-related morbidity and mortality [2, 3]. For instance, Ossewaarde’s observational studies of 12,134 women showed that mortality was reduced 2% with each increasing year of age during menopause [4]. In addition, Perls’s study found that individuals from families with a history of longevity also tended to exhibit delayed reproductive aging [5]. These studies suggest that there might be a relationship between menopause age and biological aging rate, and that menopausal age is also considered to be heritable. Therefore, reliable biomarkers of menopausal age, which are currently lacking, would be useful, and might lead to further reductions in morbidity and mortality related to menopausal age.

Several studies have reported epigenetic alterations in DNA methylation in senescent cells [6-8]. DNA methylation is a reversible and heritable mechanism of epigenetic regulation that is involved in a wide variety of fundamental biological processes, including gene expression and maintenance of genomic stability [9, 10]. However, the association between DNA methylation levels and menopausal age has not been well investigated.

The measurement of methylation across repetitive DNA elements has been shown to be associated with global methylation levels [11]. Approximately 45% of the total DNA sequence consists of repetitive elements [12], among which Alu (a short-interspersed nucleotide element comprising approximately 11% of the human genome and contains 30% of its methylation sites [13, 14]) and LINE-1 (a long-interspersed nucleotide element comprising approximately 17% of the human genome [15]) are the most common and well-characterized families. Methylation levels of Alu elements and LINE-1 have been evaluated in aging models. In addition, hypomethylation of Alu but not LINE-1 has been reported as a global event in aging cells [16].

Because menopausal age is associated with biological aging and epigenetic changes are related to age, we were interested in correlating epigenetic changes with menopausal age. In addition, we aimed to investigate associations between menopausal age and the DNA methylation levels of two repetitive elements, Alu and LINE-1, in blood.

## MATERIALS AND METHODS

### Subjects

This study was performed in the department of Gynecology at the Affiliated Hangzhou People’s Hospital of Nanjing Medical University between June 2015 and February 2017.

Participants with natural menopause were recruited according to the following criteria: each individual 1) agreed to participate in the study and gave informed consent; 2) had no history of hormone replacement therapy; 3) had no history of chronic, malignant, endocrine or genetic disease; and 4) had no history of smoking or alcohol drinking.

Body mass index (BMI) in this study was calculated as weight/height^2^. Hypertension was inferred from the reported use of antihypertensive medication. Similarly, women on oral antidiabetic drugs or insulin were classified as diabetic.

We screened 300 patients for potential enrollment and confirmed 289 women (96.33%) as eligible. A total of 281 women (97.23% of those confirmed as eligible) agreed to participate in the study. Of the 281 study subjects, 161 donated a blood sample for plasma DNA extraction (plasma DNA extraction cohort), and 120 donated for serum cell-free DNA (cfDNA) extraction (serum cfDNA extraction cohort). All study procedures were approved by the ethics committee of the Affiliated Hangzhou People’s Hospital of Nanjing Medical University.

Our previous studies found that the average age of natural menopause in Chinese women is approximately 50 years old [17]. Because DNA methylation changes may be more apparent with much earlier or later menopause, we grouped women with menopausal age ≤ 48 years as the earlier menopause group, ≥ 52 years as the later menopause group, and between 48 and 52 years as the control group. Duration of menopause was calculated as present age minus the age of the onset of menstruation.

**Table 1:** Primer and probe sequences for MethyLight PCR

Gene		Primer sequence	Ref.
Alu	Forward Reverse Probe	5′-GCGCGGTGGTTTACGTTT-3′ 5′-AACCGAACTAATCTCGAACTCCTAAC-3′ 5′-6FAM-AAATAATCCGCCCGCCTCGACCT-BHQ1-3′	[11]
LINE-1 b-actin	Forward Reverse Probe Forward Reverse Probe	5′-GGACGTATTTGGAAAATCGGG-3′ 5′-AATCTCGCGATACGCCGTT-3′ 5′-6FAM-TCGAATATTGCGTTTTCGGATCGGTTT-BHQ1-3′ 5′-TGGTGATGGAGGAGGTTTAGTAAGT-3′ 5′-AACCAATAAAACCTACTCCTCCCTTAA -3′ 5′-6FAM-ACCACCACCCAACACACAATAACAAACACA-BHQ1 -3′	[11] [39]

### Plasma/Serum DNA Extraction and Bisulfite Treatment of the DNA

Three milliliters of blood were collected in EDTA tubes from the patients during the first visit. All samples were coded and frozen at -20 ºC until DNA extraction. DNA was extracted from peripheral blood leukocytes using a Simgen Blood DNA Mini Kit (Simgen, Hangzhou, China). Another 3 ml of blood was collected from participants into a sterile plain vacutainer tube for cfDNA extraction. These samples were centrifuged (1000 g for 15 minutes, followed by 16000 g for 1 minute) within 6 hours of collection to separate serum and were stored at -20 ºC until use. The BeaverBeads™ Circulating DNA Kit (Beaver, Suzhou, China) was used to isolate cfDNA from 400 μl of serum.

A total of 200 ng of DNA (concentration 10 ng/μl) was bisulfite-treated using an EpiTect™ Bisulfite Kit (Qiagen, Hilden, Germany) according to the manufacturer’s protocol. The eluted DNA sample was stored at -20 ºC.

**Figure 1.**
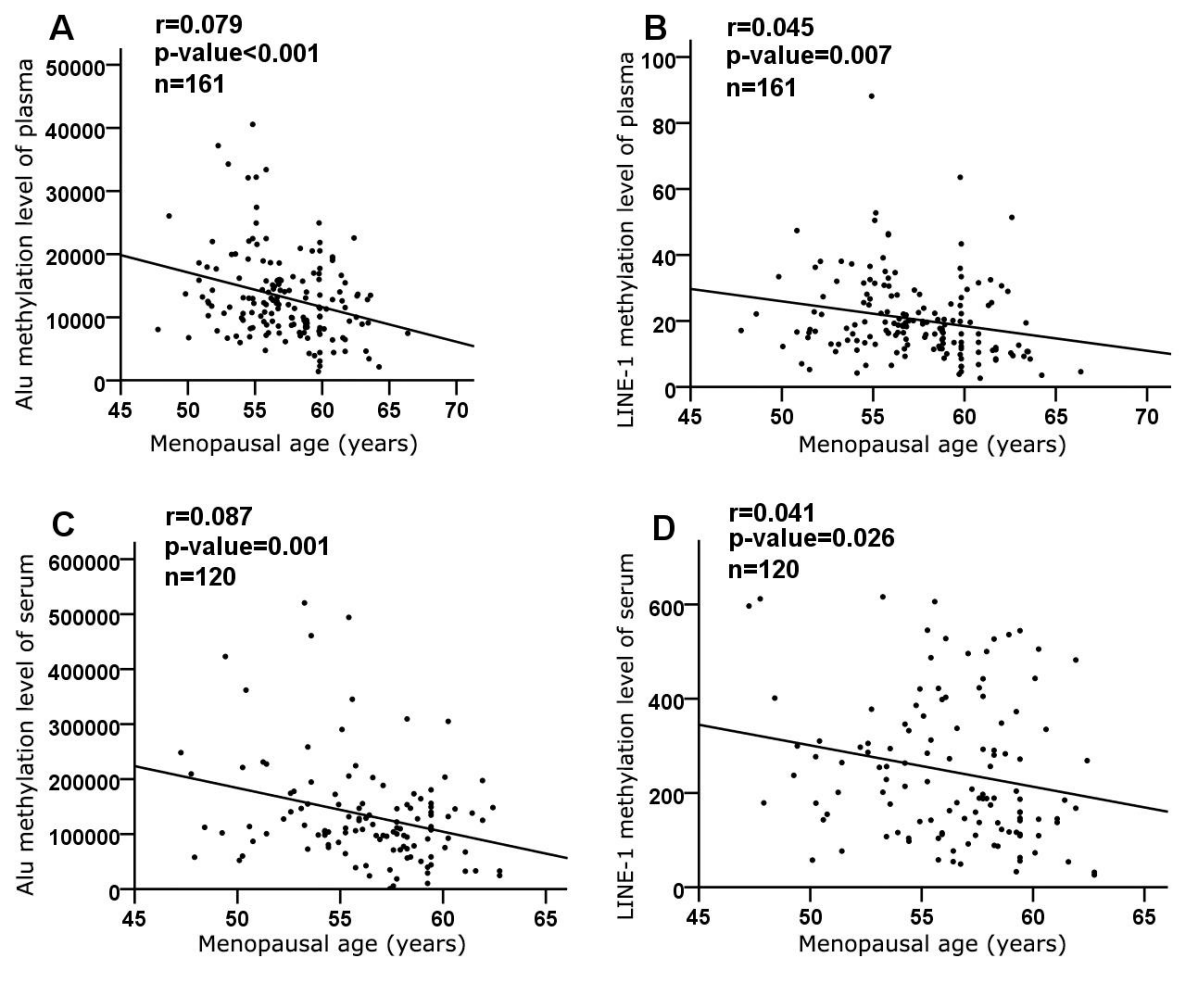
Correlation between menopausal age and methylation of Alu and LINE-1. Data were adjusted for age, BMI, hypertension, diabetes and education. Significance of correlation coefficients (r) was set at *P* < 0.05.

### Methylation measurement by MethyLight

Methylation of Alu and LINE-1 was quantified by MethyLight, a methylation-specific, probe-based, real-time PCR technique [11]. PCR primers and probes are listed in Table 1. A total of 20 μL MethyLight PCR was carried out in 10 μL of AceQ™ qPCR Probe Master mix (Vazyme, Nanjing, China), 1 pmol each forward and reverse primers, 0.1 mM TaqMan probe, 0.4 μL of ROX Reference Dye 2, 50 ng of bisulfite-treated genomic DNA, and water using the following PCR program: 95 ºC for 5 minutes and then 40 cycles of 95 ºC for 10 s followed by 60 ºC for 34 s. Assays were run on an ABI 7500 Real-Time PCR System (Applied Biosystems, Foster City, CA). Reactions were performed in duplicate with negative controls on each plate, and the average Ct value was used in the calculations. The quantified value of DNA methylation of a target gene was normalized by β-actin. Relative copy numbers of Alu and LINE-1 were calculated according to the formula [2^-ΔCt^, (ΔCt = Ct_Alu/LINE-1_ - Ct_β-actin_)].

### Statistical analysis

Continuous data were compared using the independent samples t-test. Chi-squared analysis and Fisher’s exact tests were used for categorical variables.

To evaluate the correlations between menopausal age and methylation levels of Alu and LINE-1, Pearson and Spearman correlations were used to determine associated 95% confidence intervals. Linear regression (adjusted for age, BMI, hypertension, diabetes, and education) was applied to investigate the relationship between methylation levels and menopausal age. The associations between Alu and LINE-1 methylation levels were examined using the same method. Partial correlation was used to determine 95% confidence intervals.

The methylation levels of Alu and LINE-1 in the three groups were expressed as medians with interquartile values, and the Kruskal-Wallis test was used to make nonparametric comparisons between the groups. The Mann-Whitney U-test was used as a further comparison between each pair of groups.

We used the SPSS 17.0 statistical software (SPSS, Chicago, IL, USA), and P < 0.05 was considered to indicate a significant difference. All tests were two-tailed analyses.

## RESULTS

### Characteristics of the study population

Table 2 shows the characteristics of the 161 participants whose blood was used for plasma DNA extraction. The mean menopausal ages of the earlier menopause group, later menopause group, and control group were 46.08, 53.47, and 49.98 years, respectively (P < 0.001). A total of 120 participants donated blood for serum DNA extraction. The mean menopausal ages of the earlier menopause group, later menopause group, and control group were 45.63, 53.17, and 49.99 years, respectively (P<0.001, Table 3). Overall, no significant differences were observed in either cohort among the three groups in mean age, BMI, hypertension, diabetes or education.

**Table 2:** Participant characteristics of plasma DNA extraction cohort.

	Earlier menopause group (n=60)	Later menopause group (n=52)	Control group (n=49)
Age (years)	56.47 ± 8.44	57.00 ± 5.85	58.33 ± 7.96
Menopausal age (years)	46.08 ± 2.00	53.47 ± 1.74	49.98 ± 0.81
BMI (kg/m^2^)	23.46 ± 3.08	24.35 ± 3.48	23.97 ± 3.44
Hypertension [n (%)]	11 (18.3%)	11 (21.15%)	13 (26.53%)
Diabetes [n (%)]	3 (5.00%)	5 (9.62%)	5 (10.20%)
Education [n (%)]			
Less than middle school	20 (33.33%)	22 (42.31%)	23 (46.94%)
Middle school	32 (53.33%)	25 (48.08%)	21 (42.86%)
More than middle school	8 (13.33%)	5 (9.62%)	5 (10.20%)

Statistical comparison: Student’s t-test: age, menopausal age and BMI. Chi-squared: hypertension, diabetes, and education. Data were expressed as the mean ± SD.

### Association between menopausal age and methylation levels of Alu and LINE-1

First, we evaluated the relationship between menopausal age and methylation levels of Alu and LINE-1. The DNA methylation levels of both Alu and LINE-1 were inversely correlated with menopausal age in the plasma DNA cohort (r = 0.079, *P* < 0.001 for Alu; r = 0.045, *P* = 0.007 for LINE-1) (Fig. 1A and B) as well as in the serum DNA cohort (r = 0.087, *P* = 0.001 for Alu; r = 0.041, *P* = 0.026 for LINE-1) (Fig. 1C and D). We then evaluated whether the duration of menopause influenced methylation and found that there were no differences in either Alu or LINE-1 in either cohort (data not shown).

**Figure 2.**
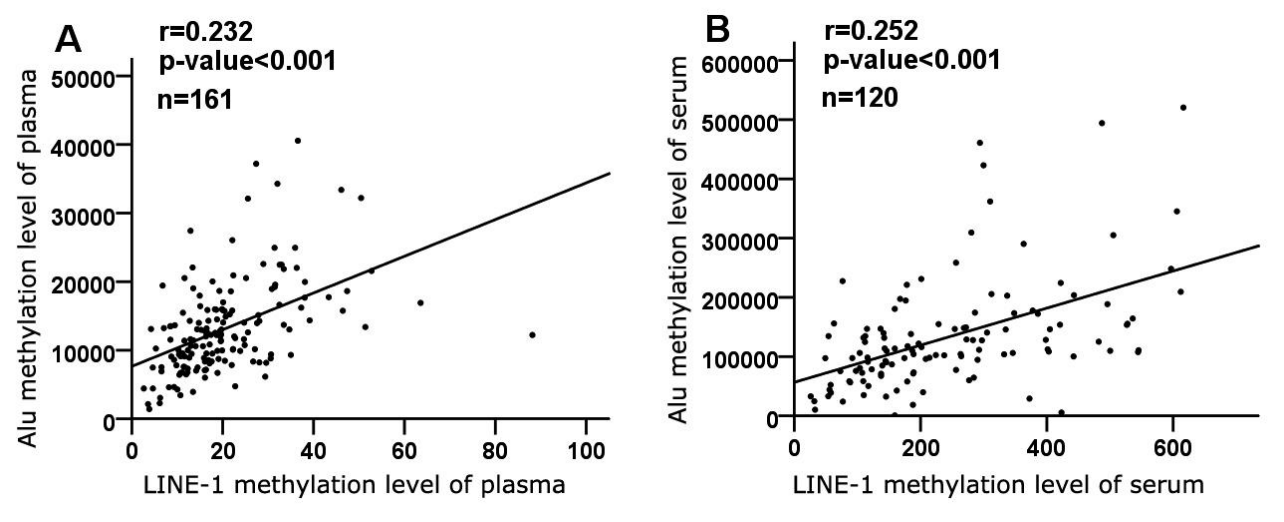
Correlation between methylation of repetitive elements. Each plot represents methylation levels of individuals, and data were adjusted for age, BMI, hypertension, diabetes and education. Significance of correlation coefficients (r) was set at *P* < 0.05.

### Correlation between Alu and LINE-1 methylation

Because both Alu and LINE-1 were demethylated with menopausal age, we evaluated the correlations of methylation levels between Alu and LINE-1. As expected, a significant positive correlation was demonstrated in the plasma DNA cohort (r = 0.232, *P* < 0.001) (Fig. 2A). There was also a significant association between Alu and LINE-1 methylation levels in the serum DNA cohort (r = 0.252, *P* < 0.001) (Fig. 2B).

**Table 3:** Participant characteristics of serum DNA extraction cohort.

	Earlier menopause group (n=40)	Later menopause group (n=42)	Control group (n=38)
Age (years)	55.05 ± 6.48	57.45 ± 5.61	56.47 ± 6.40
Menopausal age (years)	45.63 ± 2.68	53.17 ± 1.53	49.99 ± 0.83
BMI (kg/m^2^)	23.48 ± 3.86	24.75 ± 3.31	23.94 ± 3.02
Hypertension [n (%)]	9 (22.5%)	13 (30.95%)	10 (26.32%)
Diabetes [n (%)]	2 (5.00%)	4 (9.52%)	4 (9.52%)
Education [n (%)]			
Less than middle school	19 (47.50%)	22 (52.38%)	17 (44.74%)
Middle school	15 (37.50%)	16 (38.10%)	18 (47.37%)
More than middle school	6 (15.00%)	4 (10.53%)	3 (7.89%)

Statistical comparison: Student’s t-test: age, menopausal age and BMI. Chi-squared: hypertension, diabetes, and education. Data were expressed as the mean ± SD.

### Methylation levels in different menopausal age groups

**Figure 3.**
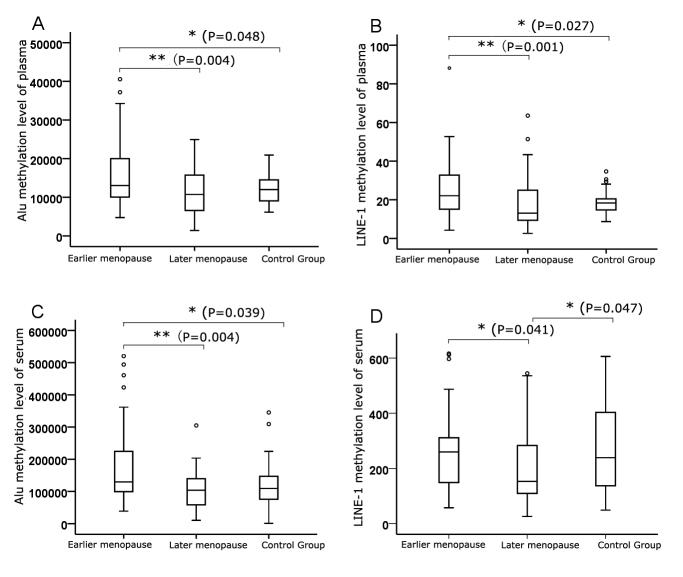
Methylation levels of Alu and LINE-1 in different menopausal age groups. Data are represented as box plots. Data were adjusted for age, BMI, hypertension, diabetes and education. * p < 0.05, ** p < 0.01.

We showed the median, minimum, and maximum Alu and LINE-1 methylation in the different menopausal age groups in Figure 3. In both the plasma and serum DNA cohorts, the levels of Alu methylation in the earlier menopause group were remarkably higher than in the later menopause group and control group (*P* < 0.01 and *P* < 0.05, respectively) (Fig. 3A and C). A trend toward lower methylation levels was observed in the later menopause group compared with the control group, but the difference was not significant (*P* > 0.05) (Fig. 3, A and C). Methylation levels of LINE-1 in the earlier menopause group, similar to those of Alu, were significantly higher than in the later menopause group and control group in the plasma DNA cohort (*P* < 0.01 and *P* < 0.05, respectively) (Fig. 3B), and methylation levels in the later menopause group showed a non-significant trend toward being lower than in the control group (*P* > 0.05) (Fig. 3B). However, in the serum DNA cohort, we observed that levels of LINE-1 methylation in the later menopause group were significantly lower than in both the earlier menopause group and the control group (*P* < 0.05) (Fig. 3D).

## DISCUSSION

In the present study, we found a significant association between the methylation levels of two repetitive elements, Alu and LINE-1, and menopausal age in both peripheral blood leukocyte DNA and serum cfDNA. Women with earlier menopause showed hypermethylation in both repetitive elements compared to healthy controls, while women with later menopause showed corresponding hypomethylation. This association did not depend on age, BMI, hypertension, diabetes, or education. These findings suggest that women with different menopausal ages have different levels of methylation of repetitive elements in blood leukocytes and serum cfDNA. In addition, our data also suggest that altered DNA methylation in leukocytes and cfDNA may represent a biomarker of different menopausal age. To our knowledge, this is the first study to investigate associations between the methylation of Alu and LINE-1 and menopausal age.

Several previous studies have found associations of Alu and/or LINE-1 hypomethylation with cancer [18-20] and aging [14, 16, 21] and hypermethylation with age-related disorders such as Alzheimer’s disease [22, 23]. Jintaridth P *et al*. [14] reported that Alu hypomethylation was associated with advanced age and lower bone mass density in postmenopausal women. However, the association between Alu methylation and menopausal age was not evaluated. Several recent studies have focused on evaluating repetitive elements as predictable and preventive markers for diseases, especially cancer [24-26]. This is also the target of our study. Modifying epigenetic markers that contribute to disease development has been proposed as a novel therapeutic approach for disease treatment and/or prevention [27-29]. This study may provide crucial information for future therapeutic/preventive measures against earlier menopause.

In our study, we found a more significant change in Alu methylation than in LINE-1 methylation. Several other reports presented similar evaluations. Bollati *et al*. [30] observed a significant decrease in average Alu methylation with aging, but this correlation with LINE-1 was weaker (only borderline significant levels). Other studies did not identify LINE-1 methylation as being associated with age [16, 31].

The mechanism responsible for Alu and LINE-1 methylation in relation to menopausal age remains unknown. In our study, Alu methylation and LINE-1 methylation were correlated, indicating that these may be dependent events in methylation related to menopausal age. Alternatively, our observation suggests that the epigenomic changes may be systemic. While we observed similar trends in Alu and LINE-1 methylation in white blood cells and cfDNA, further evaluation in other cell types is still needed to identify the mechanisms involved.

Leukocytes are composed of different proportions of distinct cell populations, such as lymphocytes and granulocytes. Previous studies have shown that subpopulations of leukocytes are correlated with global epigenetic markers. For instance, one study suggest that lymphocyte DNA might serve as a valid surrogate tissue for leukocyte DNA in the level of LINE-1 and Alu repeat methylation in women [32]. Delgado-Cruzata *et al*. [33] found that DNA methylation levels in mononuclear cells and granulocytes were statistically significantly different in the same individual for LINE-1 and Alu. However, measured by MethyLight, DNA methylation levels of Alu and LINE-1 showed no correlations between mononuclear cell and granulocyte DNA of the same individual. In other mammalian leukocytes, Wnuk *et al*. [34] used equine blood lymphocytes to monitor age-associated changes in repetitive sequences and showed a statistically significant decrease in global DNA methylation levels. In this study, we did not examine methylation levels of Alu and LINE-1 in different types of white blood cells. The results of this study are our first step to find an association between the methylation levels of repetitive DNA elements and menopausal age in total leukocytes. We are interested in doing further research in correlation between methylation of Alu and LINE-1 and subpopulations of leukocytes.

DNA methyltransferases (DNMTs) are the major determinants of physiological DNA methylation levels [35]. Studies have shown that there is less global genomic methylation with aging as a consequence of decreased DNMT activity [36, 37]. Richardson’s review [38] also postulated that decreased DNMT activity may be a determinant of the loss of global DNA methylation during aging. We are also interested in correlating DNMT activity changes with menopausal age. Combining the results of Alu and LINE-1, that may provide further evidence for mechanism of different menopausal age.

Several menopause-related studies have demonstrated that later menopause is correlated with increased lifespan [4, 5]. In addition, menopause is also considered an age-related change. We may infer that earlier menopause is associated with aging. Previous studies, as mentioned before, have demonstrated that Alu and LINE-1 hypomethylation is associated with aging. We would thus hypothesize that hypomethylation of Alu and LINE-1 might be associated with earlier menopausal age. However, our results showed that Alu and LINE-1 methylation levels were higher in the earlier menopause group compared with the later menopause group. Therefore, the methylation patterns associated with aging and menopausal age vary. In addition, methylation level changes with aging and menopausal age would have different cellular and physiological consequences. These changes are independent events.

The limitation of this study includes that all blood samples are taken after volunteers had undergone menopause, which makes it unclear whether the DNA hypermethylation/hypomethylation causes earlier/later menopause or is a consequence of menopausal age. Pleasingly, we found that the duration of menopause was not associated with the methylation of repetitive DNA elements. Therefore, we are inclined to believe that DNA methylation is the cause of menopausal age rather than the consequence. However, prospective cohort studies will be needed to further address this hypothesis. Another consideration in our study design is the division of three groups. The theoretical control group would be women without menopause at the same age as study group. But the difficulty is that we cannot know the exact menopausal age of women without menopause then. Age of menopause close to the average are considered as normal menopausal age. As mentioned in Materials and Methods, the average age of natural menopause in Chinese women is about 50 years. The study sample size limited our comparisons across subgroups, therefore, we classified women with menopausal age between 48 and 52 years as the control group. We will plan to collect blood samples from premenopausal women at different stages and follow up until menopause.

In summary, this study is the first to identify an association between menopausal age and methylation of two repetitive DNA elements in both peripheral blood leukocyte DNA and serum cfDNA. Further studies are needed to confirm this finding and answer the question of cause or consequence and to provide further evidence for the utility of methylation levels as a potential non-invasive biomarker of menopausal age.
